# Implementation and Integration of a Hospital-Wide Postpartum Hypertension Clinic

**DOI:** 10.1089/whr.2024.0149

**Published:** 2025-02-12

**Authors:** Aida Roman, Erika Faircloth, Joseph Tortora, Elizabeth Deckers, Melissa Ferraro-Borgida, Stephanie Saucier

**Affiliations:** ^1^ University of Connecticut School of Medicine, Farmington, Farmington, USA.; ^2^ Hartford Healthcare Heart and Vascular Institute, Hartford, Farmington, USA.; ^3^ Hartford Hospital Women’s Health Services, Hartford, Farmington, USA.

**Keywords:** hypertension, cardiovascular disease, health disparities, postpartum

## Abstract

**Background:**

Hypertensive disorders of pregnancy (HDP) are a leading cause of maternal morbidity and mortality in the United States with an increased risk for hospital readmission and cardiovascular disease. The American College of Obstetricians and Gynecologists recommends that women with severe HDP follow-up within 72-hours post-discharge after childbirth. The purpose of this study is to evaluate if a postpartum hypertension (PPHTN) clinic improves follow-up and management.

**Methods:**

Retrospective chart review of a referred cohort in a single-center, tertiary care hospital in Hartford, Connecticut. This study included women with severe HDP who were referred to the PPHTN clinic between March 2022 to February 2023. Primary outcomes were the percentage of patients seen within 72-hours postdischarge of hospitalization, percentage of patients achieving goal blood pressure (BP) (<130/80) at first and last follow-up visits, and hospital readmission rate. Secondary outcomes included the percentage of patients receiving HDP education materials, automatic BP cuff upon discharge from hospitalization, and antihypertensive medications prescribed postpartum.

**Results:**

Our cohort had 157 women with a mean age of 32 years old (19–44), mean body mass index (BMI) 32 kg/m^2^ (16–49), and were 39% White, 24% African American, and 33% Hispanic. Comorbidities included 41% nulliparity, 19% gestational diabetes, 23% HTN, 28% gestational HTN and 10% prior preeclampsia. Among the women seen in the clinic, 53% were observed within 72 hours, 28% achieved their goal BP at first visit, and 58% achieved their goal BP at subsequent visits. Hospital readmission occurred in 5% of women. Overall, 86% received HDP education and 89% had or were prescribed a BP cuff upon discharge. Lastly, 85% were discharged on antihypertensives and 60% required antihypertensive modification postpartum.

**Conclusion:**

Our initiative significantly improved the percentage of patients observed within 72 hours of discharge and facilitated longitudinal follow-up. Future analysis is needed to evaluate readmission rate reduction and the cost-effectiveness of the PPHTN clinic.

## Introduction

Hypertensive disorders of pregnancy (HDP), including gestational hypertension (gHTN), preeclampsia/eclampsia, and preeclampsia superimposed on chronic hypertension (HTN), affect approximately 16% of pregnancies in the United States in 2019 and are a leading cause of morbidity and mortality.^1,2^ One third of those with pregnancy-related death had a diagnosis of HDP.^1^ Pregnancies complicated by HDP result in an approximately 5% hospital readmission rate, making this the second leading cause of postpartum readmission and resulting in millions of dollars of increased inpatient care costs annually in the United States.^3–6^ The majority of these readmissions occur within 10 days after delivery.^7^ Postpartum HTN (PPHTN) is diagnosed in almost 50% of patients after a pregnancy complicated by HDP and occurs *de novo* in up to 12% of women without antepartum HTN.^8–9^ Women with HDP have a 2.4-fold increase in subsequent chronic HTN and receive a diagnosis of chronic HTN 10 years earlier than in women with normotensive pregnancies.^10^ HDP are associated with a 15.3% increased risk of downstream cardiovascular disease, which is similar to the risks associated with smoking, hyperlipidemia, and diabetes mellitus.^11^ Racial and socioeconomic disparities are evident in this population, with Black women experiencing up to 37% postpartum readmission rates in the setting of HDP.^12^

According to the American College of Obstetricians and Gynecologists, women with severe HDP should follow up within 3 days of hospital discharge.^13,14^ However, there are several barriers to meeting this follow-up timeline, including provider availability, childcare, and transportation. Barriers to access may be accentuated among socioeconomically challenged populations. Inpatient care at the time of delivery may be focused on obstetrical issues and many women do not receive adequate education on blood pressure (BP) monitoring and lifestyle modifications. Although obstetric providers have expertise in the management of gestational HTN and preeclampsia, they may be less experienced with the uptitration of hypertensive medication, chronic HTN management in the nonpregnant population, and the subsequent downstream cardiovascular risk associated with HDP. Collaboration with cardiology specialists may help provide a more intensive approach to postpartum HTN management and allow for ongoing longitudinal cardiovascular care for this at-risk population.

The increasing incidence of HDP and mounting evidence of their long-term cardiovascular consequences highlight the need for postpartum transition clinics, such as the design by Celi et al., which are able to improve maternal morbidity and mortality for these patients.^15^ These transition clinics create an important connection between patients and cardiologists to allow for more aggressive long-term risk factor monitoring and modification. To minimize barriers to access, virtual visits for BP monitoring have been implemented in other institutions with good results and reduction in readmissions.^16^

**FIG. 1.**
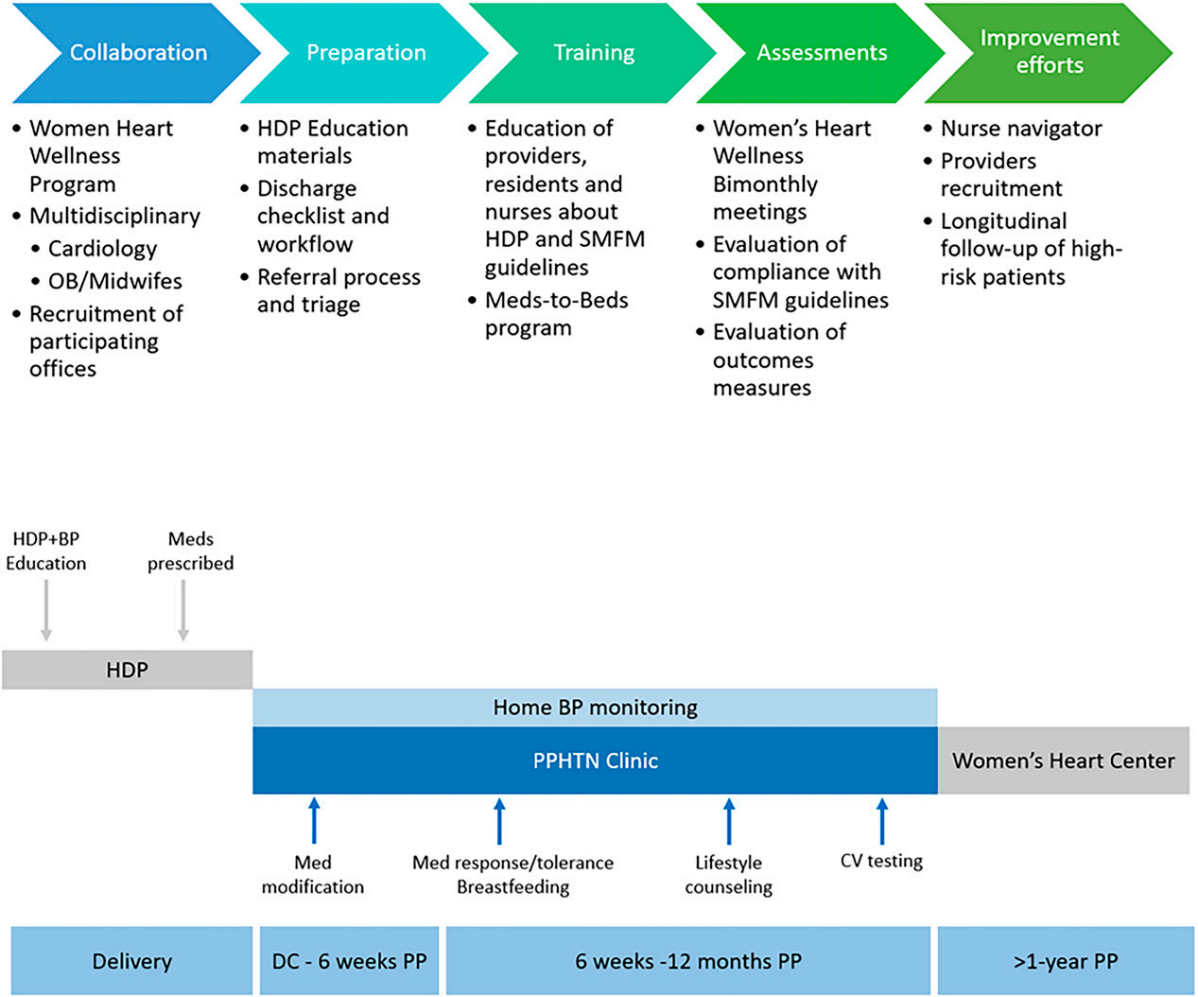
PPHTN framework. HDP, hypertensive disorders of pregnancy; SMFM, Society for Maternal-Fetal Medicine, BP: blood pressure; PPHTN, postpartum hypertension; DC, discharge; PP, postpartum; CV, cardiovascular.

The Hartford Healthcare Postpartum Hypertension Clinic was implemented in March 2022 at Hartford Hospital, a tertiary care center serving the population of greater Hartford County, Connecticut. Patients admitted with severe HTN or preeclampsia, either at the time of delivery or readmitted postpartum, were referred for early follow-up visits within 72 hours of hospital discharge. Patients were given the option of a virtual telehealth visit or an in-person visit. The general framework of the PPHTN clinic includes a comprehensive multidisciplinary program, preparation of the program’s materials, teaching of staff, and continuing evaluation of successes and limitations with ongoing improvements to the program. Following delivery and prior to discharge from the hospital, patients receive HDP and BP education along with appropriate medication prescriptions. Patients are subsequently seen in the PPHTN clinic with medication adjustment and home BP monitoring. This framework is shown in Figure 1. Our goals were to increase access to specialized providers for the management of PPHTN, improve the education about lifestyle modifications for this patient population, reduce hospital readmissions for PPHTN, and establish long-term cardiology follow-ups to allow for better downstream cardiovascular risk reduction. We present here a review of our methods and outcomes as a proof of concept for PPHTN clinics.

## Methods

### Clinic description

Patients identified as meeting criteria for severe HDP (diagnosis of severe HTN [≥160/110 mmHg], preeclampsia, severe preeclampsia, HELLP, or eclampsia) were referred to the PPHTN clinic on the day of hospital discharge. The clinic is composed of a team of cardiologists and cardiology advanced practice providers spread across several group practices within the Hartford HealthCare system. All referrals went to a central work queue and patients were triaged to geographically appropriate offices for scheduling. In-person, telehealth, or phone visits were scheduled within 72 hours of hospital discharge.

Prior to discharge, the patients received education about HDP including proper techniques for BP measurement and instructions to check and record BP readings twice per day. A printed brochure designed for the PPHTN clinic was provided to the patient at the time of discharge. These were available in both English and Spanish. BP monitors were either prescribed or provided directly to patients before discharge using the Meds-to-Beds program in collaboration with the Hartford HealthCare Community Pharmacy. The Meds-to-Beds program functions when a formal order for a BP cuff is sent from the inpatient team to the pharmacy and the BP cuff is subsequently brought to the patient’s bedside by the pharmacy staff on the day of discharge. This ensures that the patients are discharged with an automatic BP cuff so they can monitor their BP at home.

**FIG. 2.**
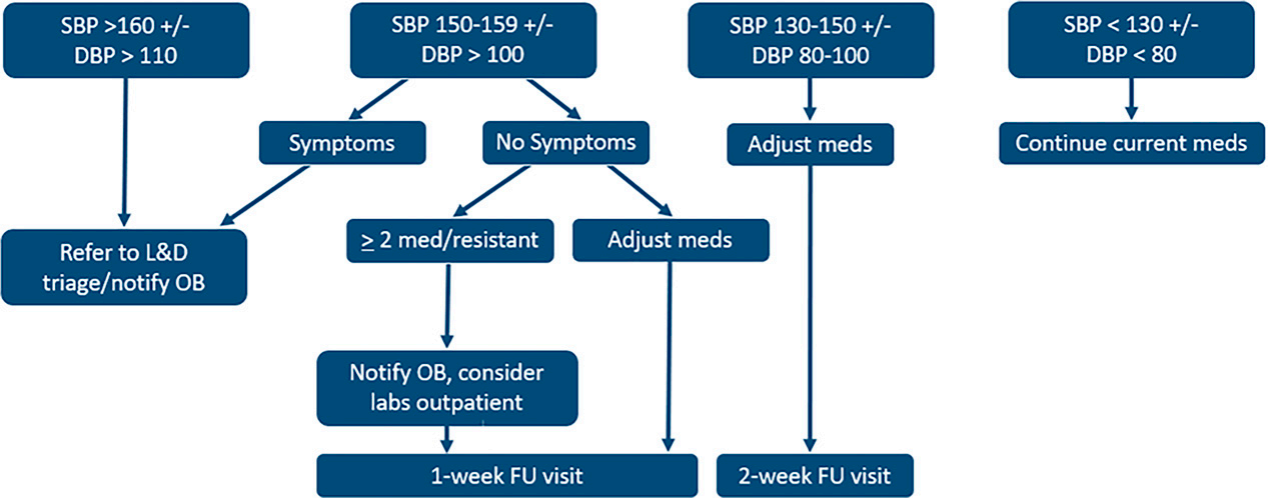
PPHTN clinic algorithm. SBP, systolic blood pressure; DBP, diastolic blood pressure; L&D, labor and delivery; OB, obstetrics; FU, follow-up.

Antihypertensive medications were prescribed prior to discharge at the discretion of the obstetrics team. Patients were scheduled for a 72-hour follow-up or for the next available appointment. Time of initial scheduling and frequency of visits were determined by a BP control algorithm (Fig. 2). Patients were also scheduled for a 6–12-month follow-up with a cardiologist to establish long-term care for cardiovascular risk reduction. Women who require follow-up for more than 12 months were transitioned to the Women Heart Wellness Program. The Women’s Heart Wellness Program is based in cardiology offices and patients are seen by either a cardiologist or cardiology advanced practice provider. The patients who are discharged to the care of primary care are those who do not require additional BP management. Those who require ongoing treatment of their HTN or other cardiology risk factors continue care with the cardiology team.

During the patients’ initial visit at the PPHTN clinic, their BP logs, antihypertensive medication regimen, and cardiac symptoms were reviewed. Patients were educated on recognizing the warning signs of preeclampsia and counseled on healthy lifestyle changes to reduce future risk of heart disease. Based on standard practice and provider preference, antihypertensive medications were optimized and further cardiac testing was ordered if deemed necessary based on the symptoms elicited by the patient.

If the patient’s BP was <130/80 mmHg, the patient was instructed to either continue current management or reduce medication dosages, if appropriate. All patients were asked to continue to monitor their BP twice a day (once in the morning and once in the afternoon), at home, between visits. If the patient’s BP was in the range of 130–150/80–100, antihypertensive medications were initiated or uptitrated and they were instructed to continue monitoring BP. If the BP was >150/100 and the patient was asymptomatic, medications were uptitrated or added. If the BP was found to be >150/100 and the patient was symptomatic, the patient was sent to Labor and Delivery at Hartford Hospital for evaluation and the cardiology provider notified the obstetric provider on-call. Patient characteristics such as breastfeeding, tolerance to medication, and desire for pregnancy in the future, as well as provider preference, were main factors influencing the antihypertensive regimen choice.

### Data collection

Women who were referred to the PPHTN clinic were included in our initial cohort. These women were diagnosed with HDP, defined as having a diagnosis of severe HTN (greater than or equal to 160/110 mmHg), preeclampsia, severe preeclampsia, HELLP, or eclampsia. Patient baseline characteristics were collected including age, race, ethnicity, BMI, type of insurance, prenatal care, nulliparous, and comorbidities (smoking, diabetes, gestational diabetes, prior HDP, and cardiac disease). In addition, mode of delivery, complications of delivery, and antihypertensive medication regimen upon discharge and at outpatient follow-up were collected. All patient data were collected from electronic health records (EHR) and were reviewed for accuracy and completeness. Patients who were not referred, followed up at the PPHTN clinic, or who delivered at other facilities were excluded. This study received an exempt status from the Hartford Healthcare Institutional Review Board.

The primary outcomes measured were the number of patients seen postdischarge within 72 hours, postpartum hospital readmissions within 6 weeks postpartum, and goal BP achieved (<130/80) at initial and last follow up visits. The secondary outcomes tracked were the percentage of patients receiving appropriate instructions and education materials upon discharge, the percentage of patients prescribed automatic BP cuffs, BP changes from discharge to follow-up, days to readmission from discharge, readmission diagnosis, and readmission length of stay. We also analyzed the number of cardiology imaging studies ordered, cardiovascular complications, number of PPHTN visits, and antihypertensive medications utilized postpartum.

## Results

From March 2022 to February 2023, 157 patients were referred to the PPHTN clinic. Demographic data for our cohort are available in Table 1. Women seen in the clinic had a mean age of 32 (range of 19–44), mean BMI 32 (16–49), and were 38.9% White, 23.6% African American, and 33.1% Hispanic. Furthermore, 52% had state-sponsored insurance and 47% had private insurance. Comorbidities included 40.8% nulliparity (preceding current pregnancy), 19.1% gestational diabetes, 22.9% HTN, 28% gestational HTN, and 10.2% had a history of preeclampsia in a prior pregnancy.

**Table 1.** Baseline Characteristics of Cohort

Demographics	Total (*n* = 157)
Age, mean (range)	32 (19–44)
BMI, mean (range)	32 (16–49)
Race, *n* (%)	
White	61 (38.9)
African American	37 (23.6)
Asian	3 (1.9)
Other	49 (31.2)
Unknown	7 (4.5)
Ethnicity, *n* (%)	
Hispanic	52 (33.1)
Non-Hispanic	98 (62.4)
Unknown	7 (4.5)
Type of insurance, *n* (%)	
Private	74 (47.1)
Medicaid/Medicare	81 (51.6)
Uninsured	1 (0.6)
Unknown	1 (0.6)
Past medical history, *n* (%)	
Former smoker	23 (14.6)
Current smoker	6 (3.8)
Diabetes	6 (3.8)
Gestational diabetes	30 (19.1)
Hypertension	36 (22.9)
Gestational hypertension	44 (28.0)
Prior preeclampsia	16 (10.2)
Pregnancy information, *n* (%)	
Nulliparous	64 (40.8)
Vaginal delivery	64 (40.8)
Cesarean delivery	92 (58.6)
Medication history, *n* (%)	
IV antihypertensives during admission	83 (52.9)
Discharged on antihypertensives	133 (84.7)

Of the 157 referrals received, 140 patients (89%) had at least one visit, 93 patients (59%) had a second visit, and 64 patients (41%) had a third visit. Out of the patients who had at least one visit, 53% (74/140) were seen within 72 hours and 79% (111/140) were seen within 5 days following discharge. Out of the patients with at least one initial visit, 28% (39/140) achieved their goal BP. Out of the patients with more than one visit, 60% (56/93) achieved goal BP at their last visit. At the 6-week postpartum mark, 5.1% (8/157) of patients were readmitted with 87.5% (7/8) of readmissions occurring within 1-week postdelivery. Readmission diagnoses included postpartum preeclampsia with severe features, postpartum preeclampsia, and hypertensive urgency. All readmitted patients received magnesium intravenously for preeclampsia protocol and most of them (5/8) required IV antihypertensives. The mean readmission length of stay was 1.75 days, ranging between 1 and 3 days (Table 2).

**Table 2.** Main Outcomes

	Pre-PPHTN initiative (*n* = 117)^a^	Post-PPHTN initiative (*n* = 157)
Characteristics, *n* (%)		
Age (mean ± SD)	32 ± 6	32 ± 6
BMI (mean ± SD)	37 ± 8	32 ± 7
Race, *n* (%)		
White	55 (44)	61 (39)
African American	25 (21)	37 (24)
Asian	4 (3)	3 (2)
Other/Unknown	36 (31)	56 (36)
Ethnicity, *n* (%)		
Hispanic	28 (24)	52 (33)
Non-Hispanic	85 (73)	98 (62)
Unknown	4 (3)	7 (4)
Primary, *n* (%)		
72-hour FU	30 (26)	74 (47)
Readmission rate	7 (6)	8 (5)
BP at goal at first visit	NA^b^	39 (25)
BP at goal at end follow-up	NA^b^	56 (36)
Secondary, *n* (%)		
HDP education	11 (9)	135 (86)
BP cuff	53 (45)	139 (89)

^a^ Pre-initiative data reported by Abunar B., Bash H., Deckers E.^17^

^b^ BP at goal at first visit and at the end of follow-up was not an outcome measure in the pre-PPHTN initiative phase.

Overall, 86% (135/157) received HDP education and 89% (139/157) had or were prescribed a BP cuff upon discharge. Mean BP reduction for the entire cohort from discharge to follow-up were −10.4 and −5.5 for SBP and DBP, respectively.

Lastly, 85% (133/157) of women were discharged from the hospital after birth on antihypertensive medications and 60% (84/140) required modification of medications postpartum. Out of the patients seen in the clinic, 48.6% (68/140) were on a single agent, 37.9% (53/140) were on two agents, and 2.9% (4/140) required three or more BP medications. Choice for antihypertensive agent used was at the discretion of the cardiology provider with the consideration of active breastfeeding and any plans for future family planning. The most common antihypertensive agent prescribed was nifedipine 69.3% (97/140), followed by labetalol 47% (66/140) and hydralazine 1.4% (2/140). The majority of patients were seen virtually. Further cardiac testing within 12 weeks postpartum occurred in six patients, which included a total of seven cardiac tests. There were four transthoracic echocardiograms ordered: two for atypical chest pain, one for volume overload, and one for a cardiac murmur on a physical exam. There was one CTA of the aorta, one exercise stress echo, and one exercise treadmill test ordered, all of which were completed for chest pain. All testing performed was found to be negative. Among all patients referred to the PPHTN clinic, the mean number of visits was 3 (SD 2.63), whereas the median number of visits was 2 (1–4).

## Discussion

In our pilot study, the implementation of a multidisciplinary initiative improved 72-hour follow-up and BP management. Our initial high show rate of 89% (140/157) was achievable with the use of virtual and phone visits, which allowed for 47% (74/157) of patients to be seen within 72 hours. Some proposed explanations for only 47% of patients being seen within 72 hours include: discharges over holidays and weekends, clinic availability, patient no-shows, patient cancellations, and inability to connect with patients to schedule initial visit. Despite these challenges, this was a significant improvement from prior reported data from our institution, which showed that only 26% of patients with severe HDP had 72-hour follow-up postdischarge.^17^ Although this prior reported data by our institution involved a quality improvement project focusing in severe and nonsevere HDP, our project mainly targeted severe HDP women who are at a higher risk of complications.

When evaluating BP management, 28% of patients achieved their goal BP at their initial visit while most patients (58%) achieved their goal BP at subsequent visits. These results highlight the care needs of this population and the significant gains with longitudinal follow-up. Furthermore, our data showed modest SBP and DBP reduction with −10.4 and −5.5 for SBP and DBP, respectively.

Although our cohort had a slight reduction in hospital readmission compared with previously reported data (5% vs. 6%), our sample mainly included patients with severe HDP who have a higher risk of readmission when compared with women with nonsevere HDP. This made our sample a higher risk population than other cohorts that typically include nonsevere and severe HDP patients. Similar to other studies, most of our patients (88%) were readmitted within 1-week postpartum and most of them were readmitted for postpartum preeclampsia and hypertensive urgency.^7,18^ This finding emphasizes the importance of close follow-up during the first weeks postpartum and high care needs of these patients.

In addition, our analysis showed a remarkable improvement in the dissemination of HDP education. The dissemination of patient education has been shown in the literature to improve patient care and reduce health care costs.^19^ Previous reported data from our institution showed that from a group of 117 patients with HDP between July and December 2020 at Hartford Hospital, 9.5% of patients with severe HDP and 0% of patients with nonsevere HDP received educational materials on discharge.^17^ After implementation of our initiative that included a nursing discharge checklist with reminders to provide BP cuffs and education, 86% of the women diagnosed with HDP received written education by the obstetrics team prior to hospital discharge.

Another strength of our study is that it harnesses the benefits of home BP monitoring. This can reduce the number of required office visits, which are often difficult to navigate for new mothers and can be a financial burden due to the need for child care and travel expenses. It also provides real world data to allow for recognition of “white coat” effect.^20^ A meta-analysis in 2013 showed that self-monitoring BP with or without clinician support led to improvement in BP control.^21^ This was further supported by a subsequent meta-analysis from 2017, showing that self-monitoring BP along with medication titration with medical providers and patient education leads to significant BP reductions, which persisted for at least a year.^22^ Moreover, a recent meta-analysis showed superiority of home BP monitoring over in-office BP readings in reducing postpartum readmission without any increase in adverse maternal, fetal, or neonatal outcomes.^20^ In our sample, 89% of patients referred to the PPHTN clinic either received a BP cuff before discharge from the hospital, had one at home, or were prescribed one. This represents a significant improvement compared with prior data from our institution, which showed that 45.2% of patients with severe HDP were ordered a BP cuff on discharge.^17^

It is also important to note that a majority of women were discharged on antihypertensives and most required uptitration of medications during clinic follow-up. The PPHTN clinic provided an opportunity for ongoing care of these patients by cardiology specialists for longer term management of HTN and cardiovascular risk reduction strategies. Proactively engaging these patients in care early allowed for these modifications of therapy, which may not have been otherwise performed.

Furthermore, the location of our hospital allowed for a racially and socioeconomically diverse cohort. Studies suggest that elevated postpartum BP, lower income, public insurance, advanced maternal age, self-reported Black race, and cesarean delivery are risk factors for postpartum readmission.^6^ Therefore, it is important to assess a diverse patient population when evaluating interventions such as the PPHTN clinic. In our study, the cohort of women was racially diverse and most had state-sponsored insurance.

This program utilized referral to cardiology practices instead of referral to primary care. The rationale for this was that patients with severe HDP have an independent risk for increased cardiovascular disease in their future. By connecting patients with a cardiology practice, they will have established longitudinal care even after the postpartum period. It was also thought that the cardiology team would have a deeper comfort with BP medication adjustments in this special population, including taking breastfeeding and future family planning into consideration. Our program also utilized telemedicine, which can be useful in resource-poor settings.

As a proof-of-concept project, there are a number of limitations. First, the sample size of our study is small. Since referrals were initiated in March 2022, it is possible that while providers were gaining understanding of the process, some eligible patients may not have been referred. The success of the program requires buy-in from multiple stakeholders including patients, cardiologists, obstetricians, office and hospital staff, and hospital administrators. These data were collected from utilization of a specific visit type in the EHR and does not reflect all patients affected by severe HDP. A decline in the number of patients who had an initial visit without a subsequent follow-up is another limitation, as there are a multitude of reasons for this decline in patients’ visits: both clinic and patient factors. Since the evaluation of the first year of data, a mandatory 12-week follow-up visit has been implemented.

Another limitation was patient-reported race data were obtained from the EHR for the demographic information. This created a limitation because the only race options were White, African American, Asian, and other. Thirty-six percent of women selected other; of these, most of them also selected their ethnicity as Hispanic. In addition, comparison of preinitiation data is limited as this came from a former quality improvement project and the methodology between projects differed. Since the initiation of the PPHTN program, a dedicated postpartum nurse navigator was hired for direct assistance with this patient population. We believe this will help with proper identification of eligible patients, streamline the referral process, and result in an increase in referrals to the program.

Second, meeting the goal of the 72-hour follow-up proved to be challenging. Dedicated spots were initially blocked in cardiology offices for these patient referrals but the increasing demand for the PPHTN clinic required that we increase the recruitment of cardiology providers to meet this need. Follow-up phone calls after discharge may provide an opportunity to engage patients, ensure they are monitoring BP at home, and encourage attendance at these visits.

## Conclusion

This paper illustrates proof-of-concept of the implementation of a PPHTN clinic. Our initiative significantly improved 72-hour follow-up and allowed for longitudinal care for women with severe HDP. A larger sample size is expected for future data analysis, which will be required to assess the reduction in readmission rates and cost effectiveness of the program.

## Abbreviations Used

BMI: body mass index
BP: blood pressure
CV: cardiovascular
DBP: diastolic blood pressure
DC: discharge
FU: follow up
gHTN: gestational hypertension
HDP: Hypertensive Disorders of Pregnancy
HTN: hypertension
L&D: labor and delivery
OB: obstetrics
PP: postpartum
PPHTN: postpartum hypertension
SBP: systolic blood pressure
SMFM: society for maternal-fetal medicine
